# Molecular diagnostics in a teacup: Non-Instrumented Nucleic Acid Amplification (NINA) for rapid, low cost detection of *Salmonella enterica*

**DOI:** 10.1007/s11434-012-5634-9

**Published:** 2013-04

**Authors:** Ryo KUBOTA, Paul LABARRE, Bernhard H WEIGL, Yong LI, Paul HAYDOCK, Daniel M JENKINS

**Affiliations:** 1 Molecular Biosciences and Bioengineering, University of Hawaii at Manoa, Honolulu, HI 96822, USA; 2 Diagenetics, Inc, Honolulu, HI 96822, USA; 3 Program for Appropriate Technologies in Health (PATH), Seattle, WA 98109, USA; 4 Human Nutrition, Food, and Animal Science, University of Hawaii at Manoa, Honolulu, HI 96822, USA; 5 Blood Cell Storage Inc (BCSI), Seattle, WA 98109, USA

**Keywords:** biosensor, assimilating probe, LAMP, DNA, food safety, molecular diagnostics

## Abstract

We report on the use of a novel non-instrumented platform to enable a Loop Mediated isothermal Amplification (LAMP) based assay for *Salmonella enterica*. Heat energy is provided by addition of a small amount (<150 g) of boiling water, and the reaction temperature is regulated by storing latent energy at the melting temperature of a lipid-based engineered phase change material. Endpoint classification of the reaction is achieved without opening the reaction tube by observing the fluorescence of sequence-specific FRET-based assimilating probes with a simple handheld fluorometer. At or above 22°C ambient temperature the non-instrumented devices could maintain reactions above a threshold temperature of 61°C for over 90 min—significantly longer than the 60 min reaction time. Using the simple format, detection limits were less than 20 genome copies for reactions run at ambient temperatures ranging from 8 to 36°C. When used with a pre-enrichment step and non-instrumented DNA extraction device, trace contaminations of Salmonella in milk close to 1 CFU/mL could be reliably detected. These findings illustrate that the non- instrumented amplification approach is a simple, viable, low-cost alternative for field-based food and agricultural diagnostics or clinical applications in developing countries.

*Salmonella enterica* is a gram negative bacterium which causes acute gastroenteritis. It is responsible for nearly half of global food-borne disease outbreaks [1], and includes serovars that cause typhoid fever [2]. In the United States alone, infection by non-typhoidal *Salmonella* results in over 19000 hospitalizations and almost 400 deaths annually [3]. *Salmonella* is often found on poultry and egg products, but it may also occur on a variety of other agricultural products including vegetables which are more likely to be consumed raw [4]. The US Department of Agriculture has estimated that Salmonella cost the country $2.7 billion in 2010 just in medical expenses, lost wages, and premature death. Losses associated with product recalls and erosion in consumer confidence in food safety are incalculable.

To help combat the risk of food-borne illnesses, poultry operations in the US are required to operate a HACCP (Hazard Analysis and Critical Control Points) plan. As part of these food safety programs producers must screen their facilities and products for Salmonella. Typically this requires shipping samples to a laboratory where they are cultured or subjected to analysis in very expensive, cumbersome laboratory instruments. Delays in obtaining test results make control and containment of food-borne pathogens extremely difficult. The main objective of this work is to develop simple diagnostic methods for *Salmonella* and other food and agricultural pathogens that can be implemented on site in makeshift facilities by typical producers and processors, thus facilitating control of disease.

Our strategy has been to focus on gene-based detection which can easily be adapted to new organisms and disease states, and which can more powerfully classify specific traits and risk factors of a pathogen. For example, gene based methods are the only rapid method for discriminating cold tolerant populations of the bacterium *Ralstonia solanacearum* [5] from cold-susceptible strains, and emerging lethal strains of *E. coli* are difficult to quickly distinguish from less harmful strains using traditional bioaffinity approaches.

The development of simple point-of-care diagnostics is an active area of research especially for biomedical applications [6,7]. Reports abound of integrated microfluidic devices for sample preparation and DNA replication by PCR [8,9]. In our work we have focused on isothermal methods- notably Loop Mediated Isothermal Amplification [10] which allows sensitive detection of pathogen-specific DNA without the tradeoffs in system complexity, power requirements, and speed that is incumbent on PCR based diagnostics. LAMP has advantages over other isothermal gene amplification technologies because it can be implemented with a single enzyme (strand displacing polymerase) and it does not require preliminary manipulations to construct a nucleic acid fragment that forms the basis for continuous isothermal replication.

While we have developed inexpensive, handheld prototype instruments to implement real-time detection of the LAMP reaction [11], even the modest power requirements (~1 W) required for these devices can be a barrier to a truly portable battery-operated system that is suitable for field settings or for clinical diagnostics in developing countries where the electrical power grid can be unreliable. Therefore, we have also been developing devices that can reliably maintain diagnostic reactions at the required temperature without instrumented temperature control. Previously, we demonstrated that the LAMP reaction can be implemented in a non-instrumented device wherein the prescribed reaction temperature is maintained by storing latent energy within a phase change material with a melting point at the desired temperature [12,13]. Thermal energy was added to the system by the exothermic hydration of calcium oxide powder, and in follow up prototypes alternative PCM materials and exothermic reagents were used to maintain nominal temperatures suitable for other representative isothermal nucleic acid amplification reactions [14]. For this report, we use the same principle for non-instrumented temperature control but use a small amount of boiling water as an energy source to facilitate cleanup and handling, storage, and disposal requirements for the highly hygroscopic CaO powder and resulting calcium hydroxide by-product. In addition, the NINA chamber was redesigned to accelerate the energy transfer into the reactions and allow for more reactions to be run simultaneously. Finally, we illustrated the use of the redesigned system for detection of DNA from *Salmonella enterica* at a wide span of ambient temperatures.

## 1 Materials and methods

### 1.1 Preparation of purified DNA standards

*Salmonella enterica subsp. enterica ser. Typhimurium* (ATCC#14028) was cultured, and DNA isolated and quantified, as described previously [11].

### 1.2 Rapid DNA extraction from culture and contaminated milk

*Salmonella* cultured as described above was used to prepare serial dilutions in 0.1% peptone water, as well as in 2% fat milk purchased from a local grocery store. To quantify contamination levels, dilutions of the cultured media were plated onto semi-selective Xyline-Lysine-Desoxycholate (XLD) agar (Catalog No. 221284, Becton Dickinson, Franklin Lakes, NJ, USA), incubated for 24 h at 35°C, and inspected for characteristic black colonies.

A rapid disposable DNA isolation platform (SNAP Cards, BCSI, Seattle WA, USA) was used to rapidly prepare samples for analysis. Briefly, 40 μL subtilisin protease reagent (BCSI) was added to 0.5 mL milk samples which were then incubated at room temperature for 30 min. The digests were then mixed with 1 mL lysis buffer (BCSI), loaded onto the SNAP cards, and left for 1 h to enable DNA to adhere to the card. Samples were then removed with a pipette, and cards were sequentially washed and DNA eluted according to the manufacturer’s protocol. For samples prepared directly from culture, the protease digestion step was omitted. To improve the detection limit in milk, some samples were loaded directly onto the cards without protease digestion or dilution in lysis buffer, and incubated at 35°C for either 8 or 24 h to enable *Salmonella* to grow out directly on the card. Following incubation these samples were removed by pipette. Then 1.5 mL of diluted lysing buffer (1.0 mL lysing buffer+distilled water) was loaded into each card, and cards held at room temperature for 1 h. Cards were then washed and DNA eluted as described above.

### 1.3 LAMP reaction and assimilating probe

LAMP reaction mixes were prepared using the same primer and probe sequences and reagent compositions as described previously [11]. Reactions were carried out in 0.2 mL PCR microtubes (Catalog No. 53509, VWR Inc., West Chester, PA, USA), using the NINA devices described below for temperature control.

### 1.4 Non-instrumented Nucleic Acid Amplification (NINA)

**Figure 1:**
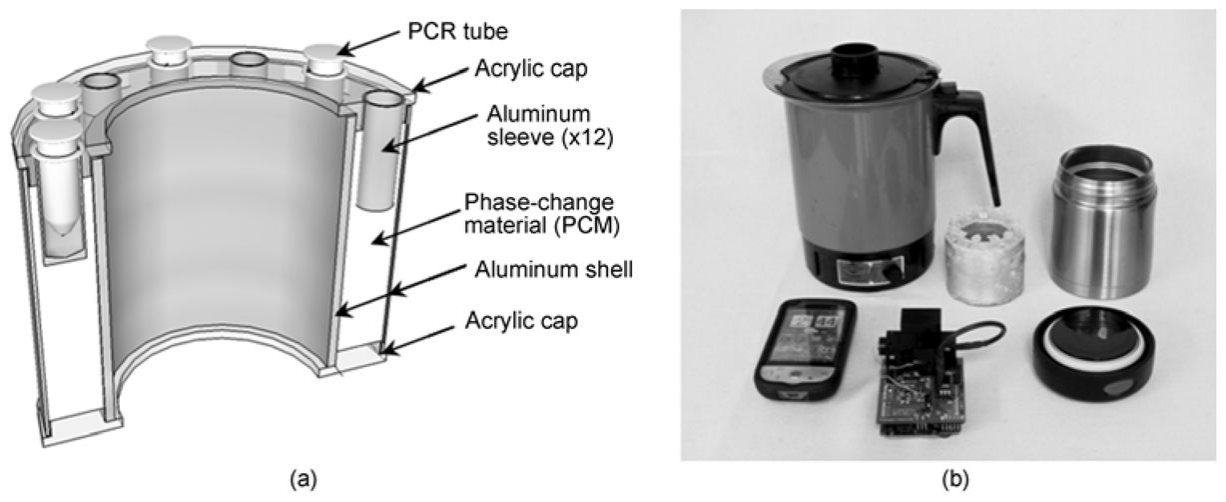
Hardware for non-instrumented nucleic acid amplification. (a) 3-D perspective cross sectional view of NINA cartridge; (b) photograph of mobile pathogen detection lab including clockwise from top left electric kettle, NINA cartridge, thermos with lid, and custom handheld fluorometer with smart phone interface.

For the redesigned non-instrumented nucleic acid amplification system, hollow cylindrical NINA cartridges (Figure 1(a)) were fabricated as an aluminum shell filled with a lipid based phase change material (PCM) with a nominal melting temperature of 65°C (PureTemp 65, Entropy Solutions Inc., Plymouth, MN, USA). Caps on the NINA cartridges were made of acrylic to prevent excessive heat transfer to the reaction tubes before the PCM reservoir reached approximate thermal equilibrium. Twelve aluminum sleeves were built into each cartridge top cap to accommodate standard 0.2 mL PCR tubes. All aluminum and acrylic parts of the cartridges were fit together with a two-component epoxy (EP31, MasterBond, Hackensack, NJ, USA) and cured overnight. Small holes were drilled in the cartridge top caps to prevent excessive pressure buildup within the cartridges due to thermal expansion of the PCM. NINA cartridges were designed with a 6.6 cm outer diameter so they could slip into a stainless steel double-walled vacuum-insulated food jar/thermos (Part #JCG300P, Thermos LLC, Rolling Meadows, IL, USA). To run a set of LAMP reactions, PCR tubes were loaded into a NINA cartridge which was inserted into a thermos. Boiling water was then added to predefined fill levels on the NINA cartridge (approximately 145 g for reactions run at or below 22°C ambient temperature, or 125 g for reactions run at 36°C) and thermos caps screwed on to maintain as much energy as possible within the NINA device throughout the course of the reaction. All of the materials required to implement NINA and subsequent endpoint detection of reactions are shown in Figure 1(b).

To evaluate the thermal performance of the NINA devices, a small hole was bored through one thermos lid through which three thermocouples made from small gage type K wire (Part Number TT-K-40-100, Omega Engineering Inc., Stamford, CT) were fixed with epoxy. These thermocouples were used to monitor temperature within a representative reaction well of the NINA cartridge as well as within the water in the cartridge shell. Thermocouple output was monitored by a meter with internal software temperature compensation (Model 189, Fluke Corporation, Everett, WA, USA), and recorded temperatures were communicated serially every 10 s to a custom application on a personal computer. Temperature profiles were recorded within three different NINA devices operated at each of three different ambient temperatures of 8, 22, and 36°C. Trials at 8°C were conducted in a walk in cold room. Trials at 22°C were conducted in an air conditioned laboratory. Trials at 36°C were conducted in a shaded area of a greenhouse under renovation, using manually controlled ventilation through a hole in the wall to regulate the ambient temperature. Prior to all NINA experiments, all materials were allowed to thermally equilibrate to the ambient temperature for at least 2 h.

Collected temperature profiles in the reaction wells were evaluated to determine time required to preheat to 61°C, average and standard deviation of temperature for 60 min after reaching 61°C, root mean squared (RMS) deviation from the design value of 65°C during the 60 min after reaching 61°C, the maximum temperature, and the temperature 60 min after reaching 61°C.

For evaluation of the performance of LAMP reactions within the NINA devices, a separate set of tests was conducted on NINA devices without temperature measurement. Reaction tubes were maintained within the NINA devices for 60 min after addition of boiling water and then removed for fluorometric analysis as described below. Triplicate reactions were run for each of a set of serial dilutions of *S. enterica* DNA at each of three temperatures (8, 22, and 36°C). NINA trials at 8 and 22°C were conducted as described above, and those at 36°C were done entirely a lab incubator (Cat #414004-618, VWR Inc) to allow more stringent control of ambient temperature. Standards ranged in DNA content from 5 fg (nominally equivalent to about 1 genome copy) to 5 ng (equivalent to approximately 10^6^ genome copies) per reaction, and negative control reactions were run with no template DNA.

### 1.5 Endpoint fluorescence detection

Fluorescence values of completed reactions were measured with the fluorometer function of a custom handheld device designed to enable real-time control and monitoring of the LAMP reaction [11]. Data from the fluorometer could be polled serially from a personal computer or wireless device such as a data phone. For comparison, fluorescence measurements were also made on the blue channel of a commercial handheld fluorometer (Quantifluor, Promega Corp., Madison, WI, USA). Existing commercial handheld fluorometers including the one we used are typically priced at about $2000 and above. In contrast, our custom fluorometer was assembled with components costing less than $200, including components required to include temperature control functionality to make a truly real-time handheld instrument.

### 1.6 Statistical analysis

Samples were classified as positive or negative for LAMP reaction based on comparison of observed endpoint fluorescence values to a threshold value. The threshold value was determined by summing the average fluorescence observed in 18 reactions with no-template DNA plus three standard deviations of the mean of these values. All fluorescence data were used to determine a best fit logistic model of the form [11]:

(1) $$F=y_{0}+\frac{a}{1+{\left(\frac{x}{x_{0}}\right)}^{b}},$$

where *F* is the relative fluorescence value, *x* is the quantity (fg) of DNA in the reaction, and *a*, *b*, *x*_0_, and *y*_0_ are empirical coefficients. Regression was implemented numerically using a commercially available software (SigmaPlot 10.0, Systat Software, Chicago, IL, USA). Detection limits were estimated from the regression results as the DNA quantity equivalent to a relative fluorescence value of three times the root mean squared error of the regression above background [12,15].

For classifying samples as positive or negative for *S. enterica* DNA, a fluorescence threshold was determined as the average plus three standard deviations of the fluorescence values observed in negative control reactions. Samples with endpoint fluorescence values above this threshold were classified as positive.

Results tended to be highly variable at low levels of contamination, so that some positively classified standards at low concentrations were excluded from the analyses to determine the best fitted curves and detection limits. The criteria for exclusion was any positively classified standard with a contamination level below the highest level at which at least 2 out of the 3 replicates classified as negative. While excluding these samples from the statistical analysis lowered the regression errors and enabled statistical detection limits to be estimated, we propose that this practice is still conservative as the detection limit is defined as the minimum level of contamination which results in statistically consistent detection.

## 2 Results and discussion

### 2.1 Thermal performance of NINA devices

**Figure 2:**
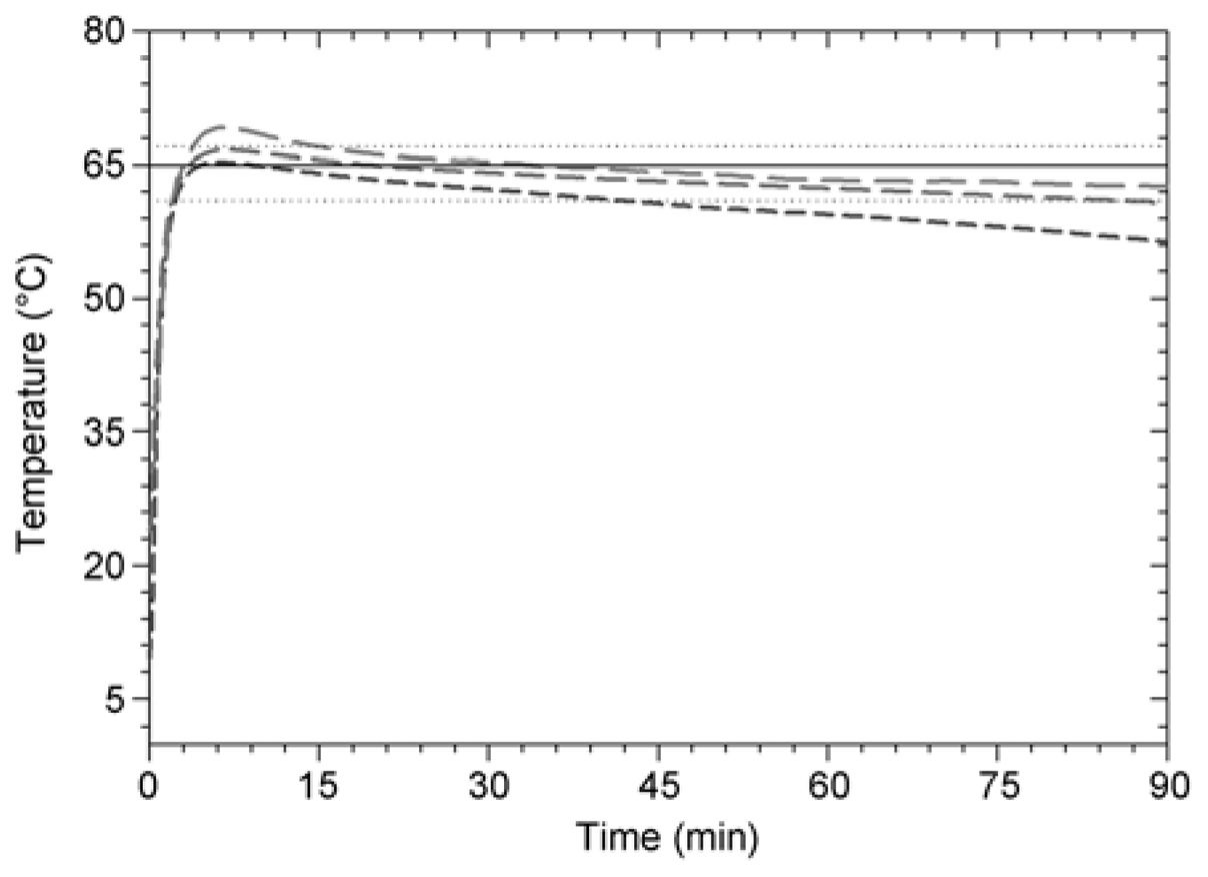
Average temperatures observed in reaction wells of NINA devices run at ambient temperatures of: 8°C (- - -); 22°C (— — —), and; 36°C (– – –). Boiling water is added to NINA devices at time=0. Horizontal lines show design temperature (—) with upper and lower bounds (···).

NINA devices generally heated reaction wells to 61°C within about 2 min of adding boiling water, and maintained the wells within the predefined specification between 61 and 67°C for at least an hour (Figure 2; Table 2). When used at laboratory temperatures (22°C) reaction wells generally reached maximum temperatures near the 67°C specification for several minutes, and at hotter ambient temperatures (36°C) reaction wells generally reached maximum temperatures of 69°C, exceeding the defined upper specification by 2°C. These overshoots from the 65°C design temperature likely occurred as a result of local temperature gradients within the PCM during initial equilibration with the added water, as melted (higher temperature) PCM floats above the solid material. Temperature overshoots observed at elevated ambient temperatures may be diminished by designing narrower aluminum sleeves for PCR tubes that are better insulated from the outer aluminum jacket, and by doing more empirical experiments to determine an optimal (lower) amount of boiling water to add for these temperatures. While the approach of using boiling water as an energy source accelerates the time to reach design temperature, this accelerated heat transfer also results in greater temperature gradients within the PCM material and poorer overall temperature control. Trials run at 8°C ambient temperature did not result in significant overshoot of the design temperature, but failed to maintain the predefined temperature specification for an entire hour-generally falling back below 61°C after about 30 min (Figure 2; Table 2). Despite these several deviations from design specifications, NINA devices demonstrated relatively consistent temperature control over a wide range of ambient temperatures, and LAMP assay performance did not appear to be affected. In our experience, LAMP reactions using assimilating probes generally have completed within 30 min, indicating that the specification to maintain the design temperature for 60 min may be unnecessary as is borne out by the results of these experiments.

### 2.2 Validation of custom fluorometer against commercial instrument

**Figure 3:**
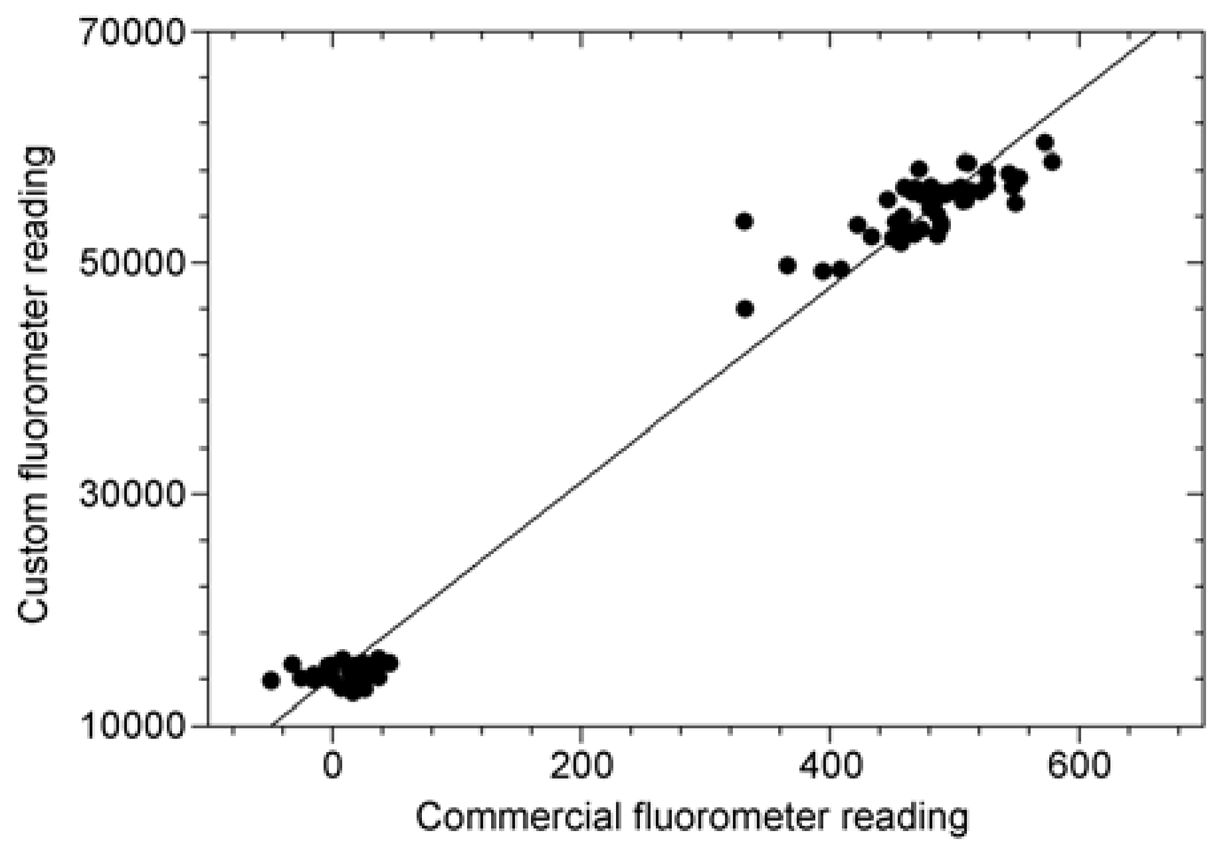
Relative fluorescence values read by custom handheld device compared to commercial fluorometer (●; —; *R*^2^=0.982; *y*=84.15*x*+14190).

Fluorescence values measured by the custom fluorometer correlated reasonably well with those measured by the commercial handheld instrument (Figure 3). In addition, classification of samples according to fluorescence values read by the real-time PCR instrument did not differ from the classifications made by the custom fluorometer (data not shown). These findings suggest that the simple handheld instrument was sufficient in range, resolution, and reproducibility to detect fluoroscence changes due to the LAMP reaction and probe.

### 2.3 LAMP amplification and endpoint detection

**Figure 4:**
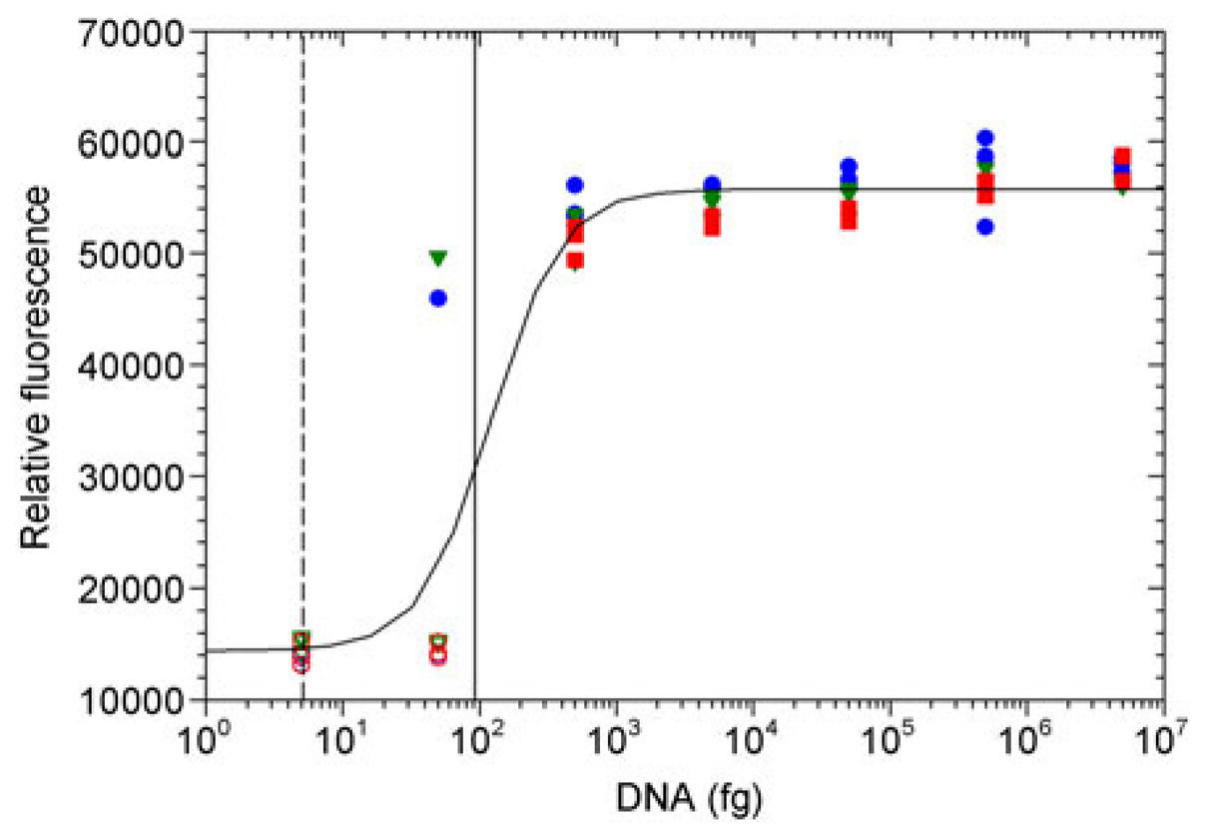
Endpoint fluorescence values for DNA standards subjected to Non-Instrumented Nucleic Acid Amplification with Assimilating Probe. Reactions were run at ambient temperatures of 8°C (blue circles), 22°C (green triangles), and 36°C (red squares), with standards classified as positive designated by filled symbols those classified as negative by open symbols. Pooled data fit a logistic curve (*a*=41400 RFU; *b*=−1.69; *x*_0_=122 fg; *y*_0_=14390 RFU; *R*^2^=0.92, RMSE=5314 RFU) resulting in a detection limit of 92 fg DNA (vertical solid line, equivalent to 18 copies of the *S. enterica* genome). A single genome copy equivalent (5.1 fg) is represented by the vertical dashed line.

Despite some minor deviations from thermal performance specifications, LAMP assays implemented within the NINA devices were robust and reliable for detecting DNA from *S. enterica* at all ambient conditions tested (Figure 4). Standard dilutions resulting in a single nominal genome copy equivalent per reaction did not result in positive amplification, consistent with low likelihoods of sampling the intact gene at these high dilution rates. Standards nominally containing about 10 genome copy equivalents were classified as positive for the LAMP reaction 22% of the time, indicating that the assay implemented in the NINA amplification approach is capable of detecting an infectious dose of 10 bacteria. None of the negative controls were classified as positive, and all samples containing 100 genome copies or more were classified correctly. Statistically, the detection limit for reliable detection was equivalent to about 18 genome copies (Figure 4), essentially the same as the detection limit for the same assay implemented in an instrumented real-time detection system [11], and significantly lower than LAMP detection based on turbidimetric analysis (~10^5^ CFU) [15], as well as the detection limit (>100 genome copies) demonstrated in the previously reported NINA device with a different primer/probe set [12]. No statistically significant differences were observed in the ability to correctly classify samples when comparing data collected at different ambient temperatures, despite relatively significant deviations in the NINA profile temperatures at the different ambient temperatures. This demonstrates that the reaction is robust at a relatively wide range of temperatures and that the polymerase is stable for at least several minutes at temperatures of 69°C. Fluorescence profiles observed using the real-time instrument [11] generally demonstrated that reactions reached completion within about 30 min, illustrating that even at the colder ambient temperatures devices were generally able to maintain the temperature design specification for durations long enough for reactions to complete. In any case, results of these experiments demonstrate that the given NINA approach is a viable option for field detection in conditions ranging from cultivated highlands and temperate areas to hot areas of the tropics.

**Figure 5:**
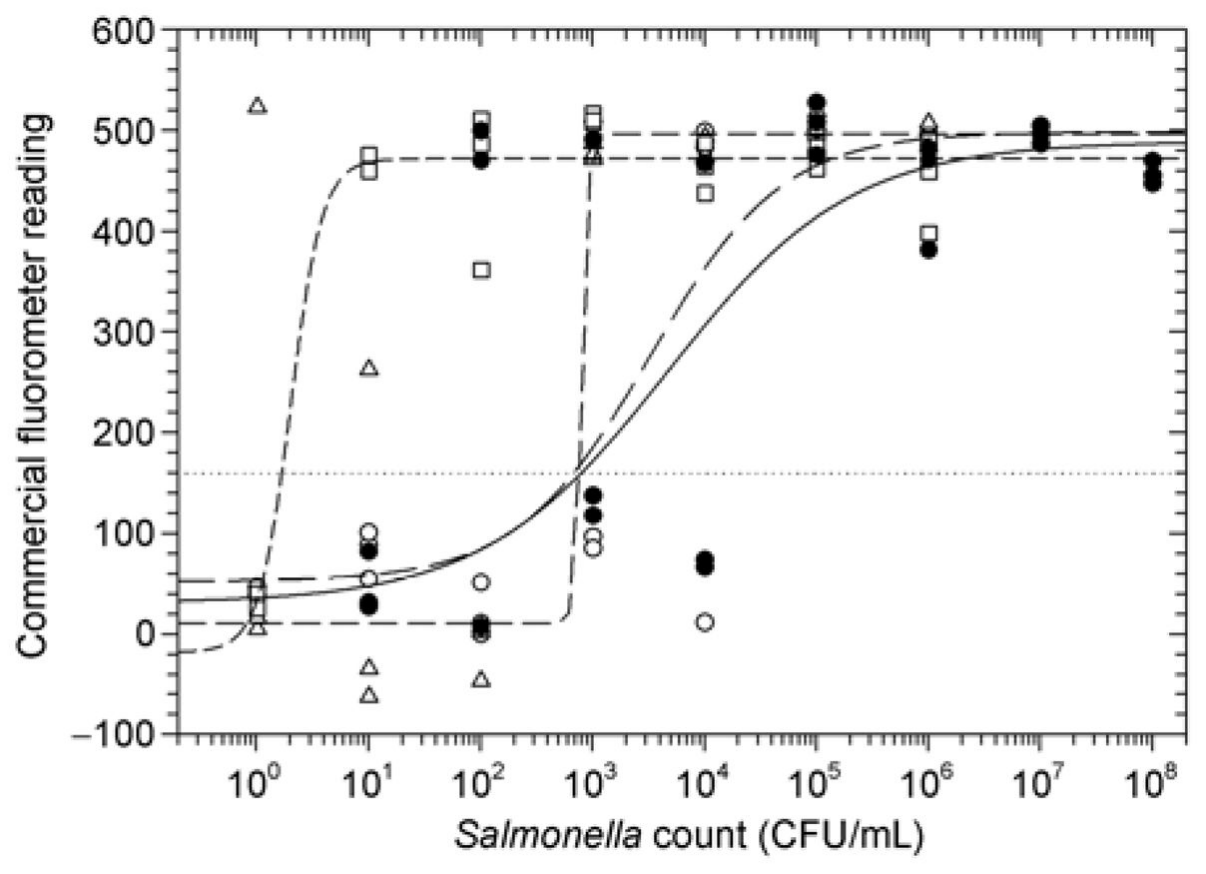
NINA assay results for DNA extracted directly from Salmonella culture (●; ———; detection limit 13000 CFU/mL), compared to results from contaminated milk samples without enrichment (○; — — —; detection limit ~28000 CFU/mL) and pre-enriched by incubating at 35°C for 8 h (Δ; ······; detection limit ~790 CFU/mL) or 24 h (□ - - -; detection limit 1.4 CFU/mL).

The endpoint non-instrumented assay was similarly successful for detection of Salmonella in culture and in contaminated milk (Figure 5). Detection limits in culture and in milk (13000 and 28000 CFU/mL respectively) were of the same order of magnitude when samples were prepared using SNAP cards, indicating that the cards successfully removed most of the inhibitory compounds from milk. Incubating samples prior to assay dramatically improved detection limits, down to about 790 CFU/mL with an 8 h incubation and to 1.4 CFU/mL with a 24 h incubation.

## 3 Conclusions and future work

Innovative new technologies were successfully demonstrated for rapid and low cost extraction and detection of pathogen-specific DNA with a minimal amount of specialized instrumentation. System performance with respect to detection was independent of a wide range of temperature conditions despite having no instrumented temperature control for the detection reaction. The detection process could potentially be used by operators in the field with a minimal amount of training by following simple instructions, and likewise the SNAP cards required no specialized equipment and could be used reliably for DNA extraction in the field.

While nucleic acid based analyses can be implemented for reliable detection directly in infected agricultural samples [15] where pathogen titres are high, media such as soil slurries, irrigation water, foods, and plant and animal tissues contain numerous inhibitors which can impair the sensitivity and detection limit of these methods. This is especially problematic when there is a low tolerance for contamination. For this work, we tested the operation of the assays for detection of *Salmonella* in milk. Detection limits were on the order of magnitude of 10^4^ CFU/mL when samples were assayed directly, which is generally consistent with results using other nucleic acid amplification techniques including PCR for real samples. In contrast however, the techniques described in this manuscript could be implemented with simple, inexpensive, non-instrumented hardware with no additional specialized equipment, with results within 3 h including sample preparation. The cost for the NINA device is approximately $50, the custom handheld fluorometer less than $200, and reagents for a single reaction about $1. Prior enrichment of samples over 24 h could improve the detection limit down to close to 1 CFU/mL, again consistent with other standard nucleic acid based techniques.

To improve detection limit reliablility without pre-enrichment, it may be possible to concentrate DNA from highly dilute, disperse pathogens in large volumes of soil, irrigation water, food, or plant and animal tissues. To address these issues we are currently investigating other commercially available systems for rapid DNA extraction, as well as our own unique approaches to rapidly process large volumes of these materials and extract purified DNA from them using simple hand-held devices. We are also coordinating additional field trials of some of these technologies for detection of a variety of pathogens in agricultural and food processing settings.

## Figures and Tables

**Table 1:** Summary statistics for thermal performance of Non-Instrumented Nucleic Acid Amplification (NINA) devices

	8°C ambient		22°C ambient		36°C ambient	
	Averageg)	Std. dev.h)	Averageg)	Std. dev.h)	Averageg)	Std. dev.h)
Time to 61°Ca) (min)	2.4	0.42	2.2	0.17	1.7	0.17
Duration >61°Cb) (min)	35.1	7.5	83.7	4.4	138.1	38.7
Avg. temp.c) (°C)	62.0	0.55	64.1	0.16	65.3	1.06
Std. dev temp.c) (°C)	1.8	0.12	1.3	0.18	1.7	0.44
RMS deviationd) (°C)	3.5	0.51	1.6	0.07	1.9	0.36
Max temp.e) (°C)	65.3	0.75	66.7	0.71	69.1	0.40
Min temp.f) (°C)	59.2	0.80	62.2	0.12	63.2	1.65

a) Time for well to reach 61°C after adding boiling water;

b) duration well is maintained above 61°C;

c) values for well temperature during 60 min period after reaching 61°C;

d) RMS deviation from 65°C design temperature during 60 min after reaching 61°C;

e) maximum observed temperature during 60 min after exceeding 61°C;

f) temperature 60 min after exceeding 61°C;

g) mean of observed parameter over all trials of devices (*n*=3 at each ambient temperature);

h) sample standard deviation of observed parameter over all trials of devices (*n*=3 at each ambient temperature).

## References

[1] Greig JD, Ravel A (2009). Analysis of foodborne outbreak data reported internationally for source attribution. Int J Food Microbiol.

[2] Brooks GF, Butel JS, Carroll KC (2007). Enteric gram-negative rods (enterobacteriaceae). Melnick & Adelberg’s Medical Microbiology.

[3] United States Centers for Disease Control and Prevention (2011). CDC estimates of foodborne illness in the United States.

[4] Sant’ana AS, Landgraf M, Destro MT (2011). Prevalence and counts of *Salmonella spp*. In minimally processed vegetables in Sao Paulo, Brazil. Food Microbiol.

[5] Kubota R, Jenkins DM, Alvarez AM (2011). Fret-based assimilating probe for sequence specific real-time monitoring of loop mediated isothermal amplification. T Biol Eng.

[6] Weigl B, Domingo G, LaBarre P (2008). Towards non- and minimally instrumented, microfluidics-based diagnostic devices. Lab Chip.

[7] Yager P, Domingo GJ, Gerdes J (2008). Point-of-care diagnostics for global health. Annu Rev Biomed Eng.

[8] Chen DF, Mauk M, Qiu XB (2010). An integrated, self-contained microfluidic cassette for isolation, amplification, and detection of nucleic acids. Biomed Microdevices.

[9] Lui C, Cady NC, Batt CA (2009). Nucleic acid-based detection of bacterial pathogens using integrated microfluidic platform systems. Sensors.

[10] Notomi T, Okayama H, Masubuchi H (2000). Loop-mediated isothermal amplification of DNA. Nucleic Acids Res.

[11] Jenkins DM, Kubota R, Higashimaru D (2011). Low-cost handheld device for sequence-specific real-time lamp-based detection of *salmonella enterica*. Biosens Bioelectron.

[12] Kubota R, LaBarre P, Singleton J (2011). Non-instrumented nucleic acid amplification (NINA) for rapid detection of *Ralstonia solanacearum* race 3 biovar 2. T Biol Eng.

[13] LaBarre P, Gerlach J, Wilmoth J Non-instrumented nucleic acid amplification (NINA): Instrument-free molecular malaria diagnostics for low-resource settings.

[14] LaBarre P, Hawkins KR, Gerlach J (2011). A simple, inexpensive device for nucleic acid amplification without electricity-toward instrument-free molecular diagnostics in low-resource settings. PLoS One.

[15] Kubota R, Vine BG, Alvarez AM (2008). Detection of *ralstonia solanacearum* by loop-mediated isothermal amplification. Phytopathology.

